# GPA Peptide Attenuates Sepsis-Induced Acute Lung Injury in Mice via Inhibiting Oxidative Stress and Pyroptosis of Alveolar Macrophage

**DOI:** 10.1155/2021/5589472

**Published:** 2021-12-28

**Authors:** Yukun Liu, Yongsheng Zhang, Quanrui Feng, Qinxin Liu, Jie Xie, Hui Li, Fan Yang, Xinghua Liu, Wei Gao, Xiangjun Bai, Zhanfei Li, Yuchang Wang

**Affiliations:** ^1^Department of Plastic Surgery, Tongji Hospital of Tongji Medical College, Huazhong University of Science and Technology, Wuhan 430030, China; ^2^Trauma Center/Department of Emergency and Traumatic Surgery, Tongji Hospital of Tongji Medical College, Huazhong University of Science and Technology, Wuhan 430030, China

## Abstract

Acute lung injury (ALI) has been known to be a devastating form of respiratory infection and an important contributor to mortality in intensive care, due to its lacking of effective treatment. Inflammation, oxidative stress, and pyroptosis are associated with multiple kinds of inflammatory diseases such as ALI. It is commonly accepted that Gly-Pro-Ala (GPA) peptide regulates oxidative stress and pyroptosis in different kinds of inflammatory diseases. Our study is aimed at exploring the regulatory function and protective effects of GPA peptides on ALI. In the current study, the cecal ligation and puncture (CLP) technique was used to evoke sepsis in mice, and GPA peptide was administered intraperitoneally with different concentrations (50, 100, and 150 mg/kg) after CLP. Histopathological changes and the ratio of wet-to-dry in lung were recorded and analyzed. We also investigated the level of oxidative stress, inflammation, and pyroptosis. Results showed that GPA peptide significantly ameliorated CLP-stimulated lung tissue injury, impeded proinflammatory cytokine release, and reduced inflammatory cell infiltration. Additionally, GPA peptide suppressed oxidative stress and caspase-1-dependent pyroptosis in alveolar macrophages. Furthermore, our study showed that the GPA peptide prevents alveolar macrophage from undergoing pyroptosis by attenuating ROS. In conclusion, results demonstrated that GPA peptide has protective effects in CLP-stimulated ALI by inhibiting oxidative stress as well as pyroptosis of alveolar macrophage.

## 1. Introduction

Acute lung injury (ALI), a frequent adverse consequence of sepsis, is a key contributor to morbidity and mortality in critical care [1]. ALI is an acute inflammatory lung disease, characterized by excessive lung infiltration of macrophages and neutrophils and a dramatic “cytokine storm,” which results in excessive lung damage [1]. Although overwhelming studies tried to explore the mechanism of ALI, the pathogenesis of the disease has yet to be fully understood. Accordingly, no effective drugs or therapies have been found for the treatment of ALI. To resolve this, the pathogenesis of ALI should be explored to increase the likelihood of clinical treatment.

The oxidative stress and pyroptosis perform an instrumental function in the pathogenesis of ALI, in which the alveolar macrophages (AMs) serve as immune surveillance in the first layer of protection against lung infection [2, 3]. Pyroptosis, which may be induced by a variety of pathogenic stimuli, can cause a fast rupture of the plasma membrane and the production of inflammatory cytokines including interleukin IL-18 and (IL)-1*β* [4]. Alveolar macrophages serve as the sentinel cells to detect infection and host defending in lung [5]. Overactivation of AMs pyroptosis leads to the release of a high number of proinflammatory cytokines, ensued by the disruption of alveolar epithelial structure and lung homeostasis [5–7]. Recent evidence suggested that caspase-1-dependent pyroptosis of AMs, a proinflammation cell death, plays a particularly important part in the progression of ALI, and inhibiting pyroptosis was considered to be a potential therapeutic target to treat ALI [5–7]. Therefore, the ALI treatment approaches have predominantly concentrated on preventing pyroptosis and inhibiting oxidative stress.

Gly-Pro-Ala (GPA) peptide, extracted from gelatin hydrolysate of fish skin, has been proven to have antioxidant and anti-inflammatory effects either in vivo or in vitro [8]. Recent literature demonstrated that Nur77 mitochondrial translocation is induced by GPA peptide, which impedes inflammation and oxidative stress by promoting autophagy subsequently [9]. Moreover, GPA peptide was also discovered as a potential therapy in DSS-induced colitis through blocking ROS and mtDNA production along with inhibiting pyroptosis in macrophages and the activation of NLRP3 inflammasome [10]. We evaluated inflammation, oxidative stress, and pyroptosis in septic mice using a well-established cecal ligation and puncture (CLP) paradigm to determine how GPA peptide affected sepsis-stimulated ALI. We sought to elucidate the mechanism through which GPA peptide modulates lung injury in CLP-stimulated septic mice.

## 2. Materials and Methods

### 2.1. Animals

The mice were kept in a temperature-controlled room with a cycle of 12-hour light and 12-hour darkness and fed with regular mouse laboratory chow and unrestricted access to water. Approval of all experiments and animal care was provided by the Animal Ethics Committee of Tongji Hospital, Tongji Medical College, Huazhong University of Science and Technology.

### 2.2. CLP Model of Sepsis

The establishment of sepsis-stimulated ALI models was done as described previously [11, 12]. Succinctly, pentobarbital sodium (40 mg/kg) was administered intraperitoneally (i.p) to anesthetize the mice. The ligated cecum was penetrated and punctured with sterile needle no. 7 at the center of the distal end and pushed the cecum back to the abdominal cavity. The abdominal wall was stitched in 2 layers, and 1 mL 0.9% sodium chloride solution was administered subcutaneously for fluid resuscitation. The mice in the sham cohort were operated on using a similar protocol but without the CLP process. Finally, subcutaneous injections of 50 mL/kg saline were used to revive the mice.

### 2.3. Experimental Protocols

Briefly, C57BL/6 female mice were categorized randomly into six cohorts. (1) sham cohort, (2) GPA peptide cohort (150 mg/kg), (3) CLP cohort, (4) CLP+GPA peptide (50, 100, and 150 mg/kg) cohort. GPA peptide was procured from Top Peptide Biotechnology Co., Ltd. (Shanghai, China). The dosage of GPA peptide was chosen based on prior studies [9, 13]. Administration of GPA peptide (50, 100, or 150 mg/kg) to the GPA peptide cohort and CLP+GPA peptide cohort was done via oral gavage at one dose each day for 7 days prior to and during CLP treatment. PBS was used to dissolve the GPA peptide, while mice in the other cohorts were given PBS intragastrically. Lung histological alterations were assessed in order to determine the highly efficacious concentration of GPA peptide for additional evaluated in vivo experiments.

Subsequently, a randomization approach was used to categorize the mice into four cohorts: (1) sham cohort, (2) GPA peptide cohort (150 mg/kg), (3) CLP cohort, and (4) CLP+ GPA peptide (150 mg/kg) cohort. The mice were euthanized in a humane manner after 72 hours, and their lungs were removed for additional examination. GPA peptide was procured from Top-Peptide Biotechnology Co., Ltd. (Shanghai, China).

### 2.4. Cell Culture

The mouse monocyte/macrophage cell line J774.A1 (ATCC, Rockville MD, USA) was cultured as previously described [6]. The cells were cultured in a standard humidified incubator at a temperature of 37°C with a CO2 concentration of 5%.

### 2.5. Pulmonary Histopathology and ALI Score

Fixing of the upper section of the lung tissues was done in 4 percent paraformaldehyde, embedded in paraffin. Then, transverse sections (5 *μ*m) were cut, and staining was done with hematoxylin-eosin (H&E). A microscope (RX51, Olympus Optical Co., Ltd., Tokyo, Japan) was used to perform histopathological investigations. As reported earlier, a modified scoring method was used to accomplish histological scoring [14, 15].

### 2.6. Lung *W*/*D* Weight Ratio

The wet left lung was excised, weighed, and dried in the oven for 24 hours at a temperature of 60°C before being weighed once more when it was dry. We computed the ratio of wet to dry weight.

### 2.7. Increase in the Protein and Cells in Bronchoalveolar Lavage Fluid (BALF)

The sacrificing of the mice was done by cervical dislocation under anesthesia with sodium pentobarbital after seventy-two hours. The chest cavities were incised to expose the lungs which were then cannulated with polyethylene tubing through the trachea. Ligation of the left main bronchus was performed followed by lavaging of the right lung thrice using 0.5 mL of PBS. The Neubauer Counting Chamber was used to count the cells by trypan blue exclusions. The residue BALF was centrifugated at 1,000 × g for 5 minutes at a temperature of 4°C, and the overall protein level was determined utilizing cell-free supernatant with ELISA kits (Dakewe, Shenzhen, China).

### 2.8. MPO Activity Assay

Homogenization of lung tissue samples was done on ice using a homogenizer. An MPO determination kit (Nanjing Jiancheng Corp., Nanjing, China) was utilized to assess MPO activity as per the instructions stipulated by the manufacturer.

### 2.9. ELISA

ELISA kits (Dakewe, Shenzhen, China) were utilized to quantify the serum levels of IL-1*β*, IL-6, and TNF-a, as well as the levels of cell supernatants and BALF, as per the protocols stipulated by the manufacturer.

### 2.10. Flow Cytometry

The collected AM cells are from the BALF and were subjected to incubation with FAM-FLICA Caspase-1 reagent for 1 hr at a temperature of 37°C utilizing the FAM-FLICA Caspase Assay Kit (ImmunoChemistry Technologies, Minneapolis, MN, USA) as per the protocols stipulated by the manufacturer. The cells were then rinsed two times using PBS and subjected to staining for 5 minutes at ambient temperature in the darkness with propidium iodide (PI) staining solution. Percentages of Caspase-1^+^ PI^+^ pyroptotic AMs were evaluated utilizing flow cytometry (BD FACSCanto™ II; BD Biosciences, SanJose, CA, USA).

### 2.11. Western Blot Analysis

Homogenization of lung tissues was done at freezing temperature in RIPA buffer containing phosphatase and protease inhibitors. SDS-polyacrylamide gel electrophoresis (SDS-PAGE) was used to separate protein specimens, which were then loaded onto a PVDF membrane. After blocking the membrane with 5 percent skim milk, an Anti-GSDMD antibody (1 : 1000, ab, Abcam, UK) was applied, and the specimens were subjected to incubation at a temperature of 4°C over the night. The membranes were once again incubated for 2 hours at ambient temperature with rabbit HRP-conjugated secondary antibody and the chemiluminescent peroxidase substrate (Millipore, Boston, MA, USA) was utilized to visualize the protein bands. ImageJ software (National Institutes of Health, USA) was utilized to quantify the densitometric analysis.

### 2.12. Immunofluorescence Assay

Subsequently, the sections were subjected to incubation over the night at a temperature of 4°C with rabbit polyclonal antibody against GSDMD (1 : 200, sigma, Cambridge, MA, USA), followed by an additional incubation for 2 hours at ambient temperature with goat anti-rabbit Cy5.5-conjugated secondary antibodies (Abcam; Cambridge, UK). After that, the sections were washed and incubated for 10 min at ambient temperature with DAPI solution. A fluorescent microscope (RX51, Olympus Optical Co. Ltd., Tokyo, Japan) was used to capture the images.

### 2.13. Oxidative Stress Assessment

SOD and ROS assay kits (Nanjing Jiancheng Bioengineering Institute, China) were utilized to measure the superoxide dismutase (SOD) and reactive oxygen species (ROS) activity as per the instruction stipulated by the manufacturer.

### 2.14. Cell Viability

CCK-8 assay (Beyotime, China) was utilized to determine cell viability as per the protocols stipulated by the manufacturer. A microplate reader (Thermo Fisher Scientific, USA) was utilized to determine cell viability by measuring absorbance at 450 nm.

### 2.15. Caspase-1 Activity Detection

The colorimetric assay (Beyotime, China), which is predicated on the cleavage of the Ac-YVAD-pNA (acetyl-Tyr-Val-Ala-Asp p-nitroanilide) substrate into pNA was utilized the measure caspase-1 activity. The production of p-nitroaniline (p NA) was utilized to assess the caspase-1 activity levels.

### 2.16. Statistical Analysis

Data are articulated as the mean ± SD. The GraphPad Prism 8 (GraphPad Software Inc., San Diego, CA, USA) was utilized to execute statistical analyses. One-way analysis of variance (ANOVA) was used to determine normal distribution data, which was then ensued by the Tukey post hoc test. Nonparametric Wilcoxon tests were utilized to analyze not normally distributed data. The Kaplan–Meier technique was employed to examine survival data, and survival curves were contrasted utilizing the Gehan–Breslow–Wilcoxon test and log-rank test in univariate analysis. Statistical significance was set as *p* value < 0.05.

## 3. Results

### 3.1. GPA Peptide Protects Mice from CLP Induced ALI in Mice

**Figure 1:**
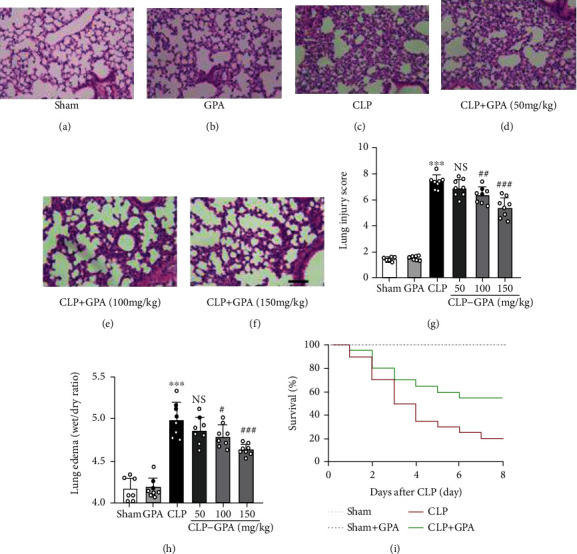
GPA peptide protected mice from CLP-induced ALI in animal models. Effects of GPA peptide on pulmonary inflammation and survival of CLP-induced septic mice. (a–f) After 72 h interventions, illustrative images of H&E staining of lung tissues from each cohort (*n* = 5/cohort). The scale bar = 50 *μ*m. (g) Lung injury score. (h) Lung edema. (i) Mice were intravenously administered with GPA peptide (150 mg/kg) in sham+GPA and CLP+GPA cohorts, and mice's survival rates were followed up for eight days. Data are shown as mean ± S.D. ^∗^*p* < 0.05, ^∗∗∗^*p* < 0.001 vs. the sham cohort; ^#^*p* < 0.05, ^##^*p* < 0.01, and ^###^*p* < 0.001 vs. the CLP cohort.

The lung architecture was investigated histologically by H&E staining to examine the influence of GPA peptide on ALI elicited by sepsis in mice. When contrasted with the sham cohort, the CLP cohort showed severe bleeding, leukocyte infiltration, and alveolar septal thickening (Figures 1(a)–1(c)). Nevertheless, GPA peptide administration considerably alleviated histopathological alterations, with the most efficacious DHM dosage being 150 mg/kg (Figures 1(d)–1(g)). In addition, the *W*/*D* weight ratio in lung, an indicator of lung edema, was shown significantly decreased in the CLP+GPA group, indicating a similar result comparing with histological grading (Figure 1(h)). These findings suggest that GPA peptide significantly attenuated lung damage in sepsis-induced ALI mice. For further studies, the 150 mg/kg dosage was selected based on these data.

To investigate the influence of GPA peptides on the survival of CLP-operated mice, we categorized them into four cohorts and followed up for eight days (Figure 1(i)). The CLP cohort's survival rate (survival: 4 of 20 mice, 20.0%) was considerably reduced as opposed to that in the sham and sham+GPA cohorts (survival: 10 of 10 mice, 100% in sham and sham+GPA cohorts, *p* < 0.001). Remarkably, the administration of GPA considerably enhanced the survival rate to 55.0% in the CLP+ GPA cohort (survival: 11 of 20 mice, *p* < 0.05).

### 3.2. GPA Peptide Reduces Lung Inflammation and MPO Activity in Lung

**Figure 2:**
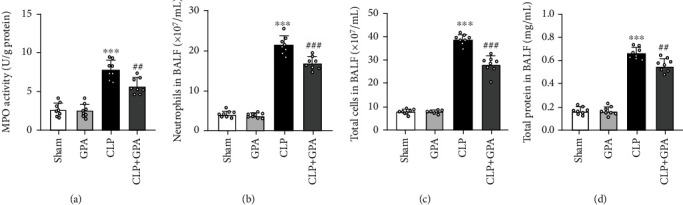
Effects of GPA peptide on MPO activity in lung tissue and lung inflammation. (a) MPO activity. (b–d) The amount of protein concentration and inflammatory cells in bronchoalveolar lavage fluid (BALF) in differentially treated septic mice at 24 hours following the CLP operation was determined to examine pulmonary vascular leakage. Data are shown as the mean ± SD. ^∗∗∗^*p* < 0.001 vs. the sham cohort; ^##^*p* < 0.01, and ^###^*p* < 0.001 vs. the CLP cohort.

BAL fluids were taken for cell counts, MPO activity, and total protein in order to additionally examine the influence of GPA peptide on inflammation of the lung. The activity of myeloperoxidase (MPO), which indicates the concentration of neutrophils in the lungs, was also considerably lowered following treatment with GPA (Figure 2(a)). The number of total cells, total protein, and neutrophils in BALF was evidently increased in the CLP cohort as opposed to the sham cohort, obviously reduced in the CLP+GPA cohort (Figures 2(b)–2(d)). Generally, these results demonstrated that GPA peptide attenuated inflammatory cell infiltration in CLP-induced ALI.

### 3.3. GPA Peptide Reduces Inflammatory Cytokine Levels in CLP-Induced ALI

**Figure 3:**
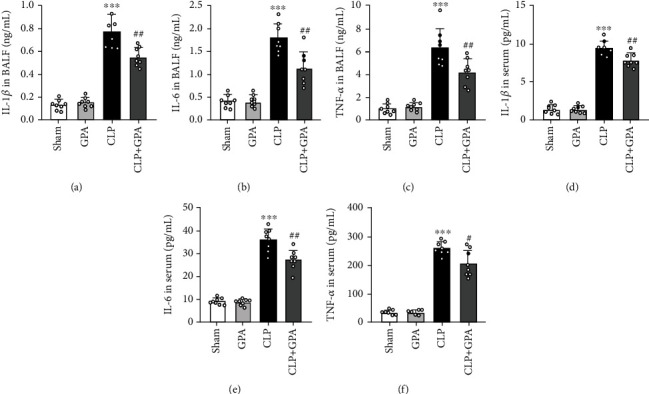
Impacts of GPA peptide on sepsis-induced inflammatory cytokine production in the BALF and serum. Measurements of IL-1*β*, IL-6, and TNF-*α* levels in BALF and serum using ELISA. Data are shown as the mean ± SD. ^∗∗∗^*p* < 0.001 vs. the sham cohort; ^#^*p* < 0.05, ^##^*p* < 0.01 vs. the CLP cohort.

TNF-*α*, IL-6, and IL-1*β* levels in BALF and serum were measured by ELISA to additionally investigate the impact of GPA peptide on the inflammation of lungs in the CLP model. There was a compelling elevation of the levels of TNF-*α*, IL-6, and IL-1*β* in BALF and serum of CLP-induced ALI which were lowered by GPA peptide therapy (Figures 3(a)–3(f)). These findings illustrated that GPA peptide had a protective effect on ALI through suppressing the inflammatory response.

### 3.4. GPA Peptide Treatment Inhibits Caspase-1-Dependent Pyroptosis in ALI

**Figure 4:**
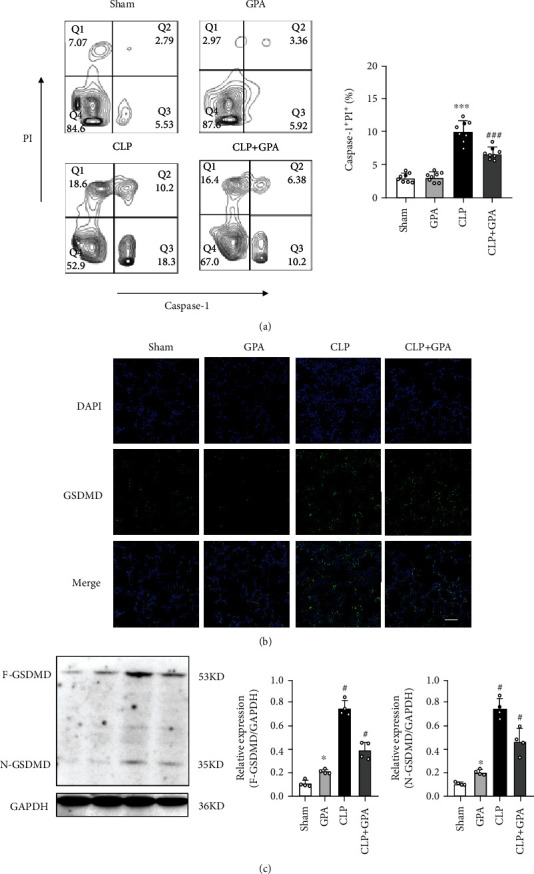
GPA peptide treatment inhibited caspase-1-dependent pyroptosis in vivo. (a) Flow cytometry analysis showing caspase-1 and PI staining. Pyroptotic cells from different groups are indicated as caspase-1^+^ and PI^+^. (b) Effects of GPA peptide on GSDMD expression. Expression of GSDMD in different cohorts was assessed using immunofluorescence. The scale bar = 100 *μ*m. (c) Immunoblot analysis of the pore-forming mediator of pyroptosis. GPA peptide markedly decreased the development of the active, cleaved N-GSDMD protein in lung tissues of sepsis mice. Data are shown as the mean ± SD. ^∗∗∗^*p* < 0.001 vs. the sham cohort; ^###^*p* < 0.001 vs. the CLP cohort.

IL-1*β* is a downstream proinflammatory cytokine of the caspase-1 activation [4]. Inflammation could be noteworthy reduced by inhibiting pyroptosis with caspase-1 specify inhibitor in LPS-induced ALI [5]. To further confirm the effects of GPA peptide in inducing macrophage pyroptosis, flow cytometry was applied to identify the caspase-1 dependent pyroptotic alveolar macrophage in vivo, and pyroptosis was marked by caspase-1^+^PI^+^. We observed that GPA peptide could significantly reduce the percentage of caspase-1^+^PI^+^ alveolar macrophage in ALI (Figure 4(a)). The pore-forming pyroptosis perforin GSDMD is yet another crucial caspase-1 target and is cleaved into its activated state by caspase-1 to form pores enhancing the swelling of cells as well as lytic cell death. Our findings illustrated that CLP substantially enhanced the generation of the active, cleaved GSDMD p30 protein in the lungs. Moreover, both GSDMD and N-GSDMD were inhibited by GPA peptide treatment (Figures 4(b) and 4(c)). In conclusion, our findings illustrated that GPA peptide suppressed the stimulation of caspase-1-dependent pyroptosis of alveolar macrophages in ALI.

### 3.5. GPA Peptide Alleviates Oxidative Stress in Septic Mice

**Figure 5:**
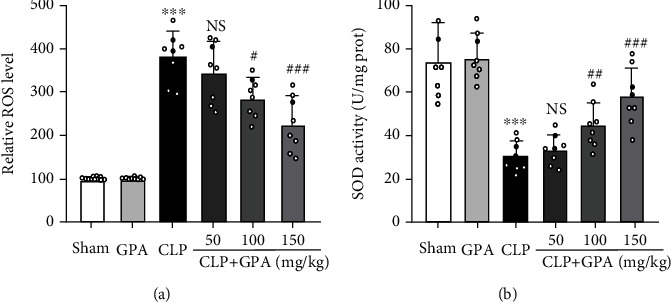
Impact of GPA peptide on oxidative stress in CLP-stimulated septic mice. (a) Commercial reactive oxygen species assay kits were used to measure the amount of ROS in mouse lung tissues. (b) Commercial SOD test kits were used to assess SOD activity in mouse lung tissues. Data are shown as the mean ± SD. ^∗∗∗^*p* < 0.001 vs. the sham cohort; ^#^*p* < 0.05, ^##^*p* < 0.01, and ^###^*p* < 0.001 vs. the CLP cohort.

ROS is known to mediate oxidative stress and has been proven to stimulate pyroptosis [16, 17]. Assessing SOD activity and ROS levels in lung tissues can be used to determine the degree of oxidative stress. GPA peptide lowered the level of ROS and improved the SOD activity in septic mice compared with the CLP cohort (Figures 5(a) and 5(b)). Our results demonstrated that GPA peptide alleviated oxidative stress in septic mice.

### 3.6. GPA Peptide Suppresses Pyroptosis and Oxidative Stress *In Vitro*

**Figure 6:**
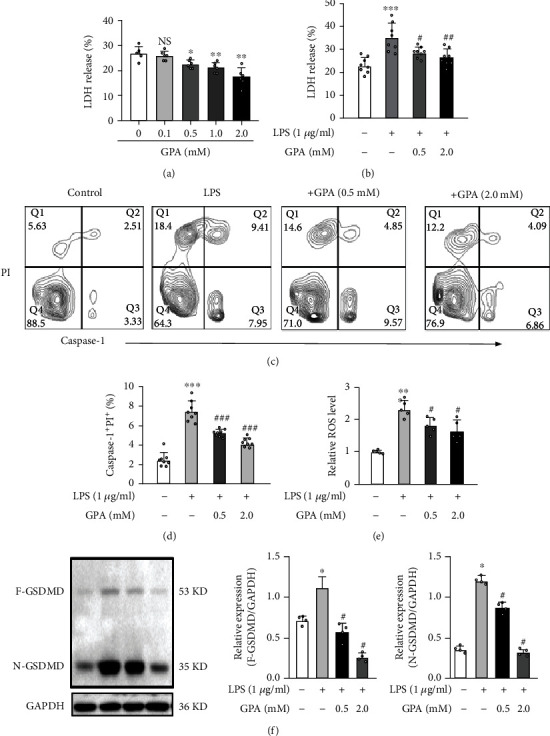
GPA peptide ameliorated pyroptosis and oxidative stress in vitro. (a) The cytotoxicity of J774.A1 cells was determined using an LDH assay after they were exposed to various GPA concentrations for 6 hours. (b) J774.A1 cells were subjected to pretreatment with various doses of GPA for 6 hours before being exposed to LPS (1 *μ*g/mL) for 3 hours, and cytotoxicity was measured using the LDH assay. (c, d) The proportion of pyroptotic cells (caspase-1^+^PI^+^) was detected using flow cytometry (FCM) after 1 h. (e) Commercial reactive oxygen species assay kits were used to assess the extracellular ROS levels. (f) Immunoblot analysis of the pore-forming mediator of pyroptosis. GSDMD cleavage was suppressed in J774.A1 cells following the LPS challenge for 6 hours. Data are shown as the mean ± SD. ^∗^*p* < 0.05, ^∗∗^*p* < 0.01, and ^∗∗∗^*p* < 0.001 vs. the control cohort; ^#^*p* < 0.05, ^##^*p* < 0.01, and ^###^*p* < 0.001 vs. the LPS cohort.

LDH was conducted to assess the optimum dosage of GPA peptides. When the concentration was 2.0 mM, our data showed that there was no evident toxicity in the normoxic conditions. Next, J774.A1 cells subjected to LPS were treated using different dosages of GPA peptide. The outcomes of LDH illustrated that 2.0 mM GPA peptide provided the optimum protective effect following LPS stimulation (Figures 6(a) and 6(b)).

In vitro, just like the inhibitory effect in vivo, the increased percentage of caspase-1^+^PI^+^ cells induced by LPS was remarkably reduced by GPA peptide treatment (Figures 6(c) and 6(d)). We also observed that LPS considerably enhanced the formation of N-GSDMD and GSDMD in J774.A1 cells (Figure 6(f)). Furthermore, treatment with GPA peptides obviously decreased ROS production (Figure 6(e)). These results indicated that GPA peptide exerts its protective effect against LPS induced through suppressing oxidative stress and pyroptosis.

### 3.7. GPA Peptide Attenuated LPS-Induced Proinflammatory Cytokines *In Vitro*

**Figure 7:**
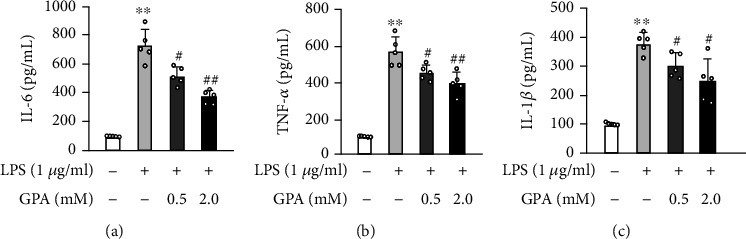
Effects of GPA peptide on LPS-stimulated TNF-*α*, IL-6, and IL-1*β* cytokine levels in J774.A1 cells. J774.A1 cells were treated using 0.5, 2.0 mM GPA peptide and subsequently cotreated with LPS (1 *μ*g/mL) for 24 hours. (a–c) GPA peptide dose-dependently alleviated LPS-stimulated TNF-*α*, IL-1*β*, and IL-6 inflammatory cytokines utilizing ELISA. Data are shown as the mean ± SD. ^∗∗^*p* < 0.01, vs. the control cohort; ^#^*p* < 0.05, ^##^*p* < 0.01, vs. the LPS cohort.

We investigated the possible anti-inflammatory benefits of GPA peptide utilizing an in vitro model of LPS-stimulated inflammation in J774.A1 cells to assess the GPA peptide's protective influence on inflammation in vitro. Treatment of GPA peptide decreased LPS-activated inflammatory cytokines which include IL-6, IL-1*β*, and TNF-*α* in a dose-dependent manner (Figures 7(a)–7(c)). These findings illustrated that GPA peptide inhibited the secretion as well as the expression of proinflammatory cytokines that include IL-1*β*, IL-6, and TNF-*α* in J774.A1 cells.

### 3.8. GPA Peptide Blocked Caspase-1 Activation by Inhibiting ROS Production

**Figure 8:**
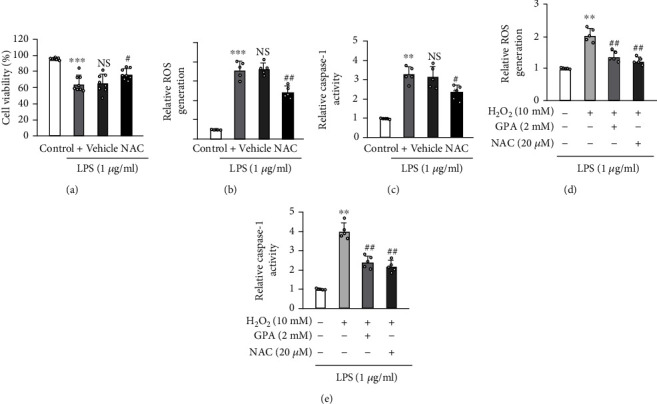
GPA peptide blocked caspase-1 activation by inhibiting ROS production. J774.A1 cells were subjected to pretreatment with 5 mM NAC for 1 hour and subsequently subjected to LPS. (a) The CCK8 assay was used to determine cell viability in the presence or absence of NAC treatment. (b) The generation of ROS was monitored, and the buildup of ROS was quantified and displayed using a bar graph. (c) Caspase-1 activity was detected. J774.A1 cells were primed with LPS for 4 hours, then treated with GPA for 6 hours before being stimulated with H2O2 for 4 hours. Levels of the ROS (d) and caspase-1 activity (e) were detected in J774.A1 cells. Data are shown as the mean ± SD. ^∗∗^*p* < 0.01, and ^∗∗∗^*p* < 0.001 vs. the control cohort; ^#^*p* < 0.05, and ^##^*p* < 0.01 vs. the LPS cohort.

To further investigate the mechanism of GPA peptide on pyroptosis, a highly efficient ROS scavenger which is N-acetyl-cysteine (NAC) was employed. We found NAC treatment suppressed ROS production (Figures 8(a) and 8(b)). Moreover, NAC treatment inhibited caspase-1 activity in J774.A1 cells (Figure 8(c)). Subsequently, we examined whether GPA peptide inhibits caspase-1 through reducing ROS by performing experiments that utilized H_2_O_2_, which contributed to higher ROS production. We found that ROS production was elevated by H2O2, however, inhibited by GPA peptide (Figure 8(d)). Correspondingly, caspase-1 activity in the supernatants upon exposure to H2O2 was reduced by GPA peptide (Figure 8(e)). Therefore, these findings revealed that GPA peptide inhibit caspase-1 activity by suppressing ROS production.

## 4. Discussion

In this study, we recognized that CLP-stimulated septic shock contributed to the evident injury in mice model, as shown by pathological alteration in morphology as well as the increased lung wet/dry ratio in lung tissues. We found that treating with GPA peptide dose-dependently decreased the morphological damage of lungs and at the same time alleviated oxidative stress injuries and inflammatory reactions. Furthermore, the administration of GPA peptide ameliorates oxidative stress and alveolar macrophage pyroptosis. Our data indicated that GPA peptide conferred protection effects against lung damage that is stimulated by septic shock in mice models.

The common pathophysiological signs of acute lung damage included intrapulmonary hemorrhage, inflammatory cells infiltration, and lung edema [1]. Recent studies have implicated that GPA peptide has a protective effect against multiple pathological factors which can result in organ injury [8–10, 13]. As illustrated by our findings, GPA peptide treatment improved LPS-induced lung damage, which is evidenced by the number of inflammatory cells in the *W*/*D* weight ratio, BALF, in addition to pathological alterations in the lungs of septic mice. Neutrophil extravasation is an initial stage of ALI's inflammatory process. The findings also indicated that CLP-stimulated ALI elevated the amount of neutrophil and inflammatory cells in BALF, which was significantly ameliorated by GPA peptide treatment. Furthermore, MPO activity, which is an efficacious measurement of neutrophil infiltration, was significantly alleviated with GPA peptide treatment.

ALI is a complex inflammatory disease, and proinflammatory cytokine release has been proven to perform a pivotal function in the pathologic process of sepsis-stimulated lung injury. In the current research, we evaluated the levels of IL-6, IL-1*β*, and TNF-*α* all of which were more enhanced in the CLP cohort both in the BALF and serum. Meanwhile, the treatment with GPA peptide significantly decreased the release of IL-6, IL-1*β*, and TNF-*α*. IL-1*β* is the product of cell pyroptosis, which was involved in the inflammatory responses associated with ALIn [5, 18, 19]. IL-1*β* can induce lung edema as well as increase pulmonary vascular permeability [18]. Collectively, these results suggested that GPA peptide exerts protective effects against CLP-induced ALI by suppressing proinflammatory cytokine production.

A variety of immune cells which include macrophages, neutrophils, mast cells, epithelial cells, and T cells are involved in acute lung inflammation [20, 21]. Recently, there has been accumulating evidence indicating that alveolar macrophages (AMs) perform critical functions in the ALI/ARDS pathogenesis. AMs are major effector cells for detecting infection and triggering host defense in lung [5]. Limiting excessive proinflammatory responses in the exudative stage via the modulation of macrophage polarization and activation has been believed as a potential therapeutic target for ALI/ARDS [22].

Cell death and inflammation are necessary characteristics in the initiation and the development of ALI [1, 5, 7]. Pyroptosis has been recognized as a lytic programmed cell death mode stimulated by inflammatory caspases (caspase-1, 4, 5, and 11) and exemplifies a key mechanism for inflammation and development of ALI [4, 7]. The gasdermin D forms holes in the cell membrane and ultimately initiates pyroptosis, which causes the mitochondria to discharge their contents [4]. Caspase-11 has been proven to control pyroptosis in endothelial cells during endotoxemia-induced lung injury [23]. Previous reports also showed that group 2 innate lymphoid cell-derived IL-9 reduces mouse lung endothelial cell (MLEC) pyroptosis in sepsis through attenuating caspase-1 activation in MLECs [24]. Recent research reports have also illustrated that AM pyroptosis performs an instrumental function in ALI. AC-YVAD-CMK, a caspase-1 specific inhibitor, may protect mice from experimental acute lung damage by blocking LPS-mediated AM pyroptosis [5–7]. Activation of the caspase-1 plays a critical role in immune defense, while excessive activation may lead to inflammatory disease happening [4, 5, 25]. Our previous studies have shown that the overactivation of caspase-1 is closely associated with the development and prognosis of sepsis [25, 26]. In this research, GPA peptide inhibits the secretion of IL-1*β*, caspase-1 activation, and GSDMD expression in CLP-stimulated septic mice, proving that GPA peptide mitigated pyroptosis in CLP-stimulated septic mice.

ROS is widely considered to be a driving force in pyroptosis [17, 27, 28]. Growing evidence has indicated that oxidative stress performs an integral function in ALI [3, 29–33]. Overproduction of ROS could intensify immune signals and therefore aggravate the tissue damage [3, 34]. SOD is the primary enzymatic antioxidant enzyme in lung as well as in the vascular wall. SOD and ROS are important indicators to assess lung damage [2]. In the current research, we discovered that sepsis contributed to the elevation in ROS level and the decrease in SOD activity in the lung of the mice model. GPA peptide was found to ameliorate acute inflammation reaction by impeding the increased ROS production and enhancing the activity of SOD. Therefore, GPA peptide may be an effective treatment to suppress ROS and prevent ALI.

Studies have addressed the effect of the ROS-NLRP3 signaling pathway on the activation of caspase-1 and organ injury [16, 17, 27, 35]. The activation of AIM2 as well as the NLR family, which includes NLRC4, NLRP3, and NALP, is thought to facilitate pyroptosis via caspase-1. Among them, the NLRP3/caspase-1 signaling has been studied most extensively [4]. In our previous study, we have found that dihydromyricetin ameliorates sepsis-stimulated acute lung injury through blocking NLRP3/caspase-1 signaling in mice models [14]. Therefore, we next explored whether ROS is involved in caspase-1-dependent pyroptosis stimulated by ALI. Our findings illustrated that NAC (a ROS scavenger) could significantly reduce the ROS level and suppress the caspase-1 activity. Furthermore, GPA peptide could suppress caspase-1 activation through inhibiting ROS production in vitro. Thus, our findings indicated that oxidative stress resulting from septic shock could have a critical function of lung injury, and GPA peptide might alleviate septic shock-stimulated AM pyroptosis by inhibiting oxidative stress for the contribution of lung protection.

To summarize, our findings showed that GPA peptide suppressed pyroptosis, inflammatory response, oxidative stress, and in septic mice, hence alleviating lung damage. The GPA peptide seems to have the prospect to be a viable adjuvant therapy approach for sepsis and acute lung damage treatment.

## Data Availability

No data were used to support this study.
